# A Fast EEG Forecasting Algorithm for Phase-Locked Transcranial Electrical Stimulation of the Human Brain

**DOI:** 10.3389/fnins.2017.00401

**Published:** 2017-07-20

**Authors:** Farrokh Mansouri, Katharine Dunlop, Peter Giacobbe, Jonathan Downar, José Zariffa

**Affiliations:** ^1^Institute of Biomaterial and Biomedical Engineering, University of Toronto Toronto, ON, Canada; ^2^Institute of Medical Science, University of Toronto Toronto, ON, Canada; ^3^Department of Psychiatry, University of Toronto Toronto, ON, Canada; ^4^Centre for Mental Health, University Health Network Toronto, ON, Canada; ^5^Krembil Research Institute, University Health Network Toronto, ON, Canada; ^6^Toronto Rehabilitation Institute, University Health Network Toronto, ON, Canada

**Keywords:** closed-loop stimulation, phase-locking, brain stimulation, tACS, EEG forecasting

## Abstract

A growing body of research suggests that non-invasive electrical brain stimulation can more effectively modulate neural activity when phase-locked to the underlying brain rhythms. Transcranial alternating current stimulation (tACS) can potentially stimulate the brain in-phase to its natural oscillations as recorded by electroencephalography (EEG), but matching these oscillations is a challenging problem due to the complex and time-varying nature of the EEG signals. Here we address this challenge by developing and testing a novel approach intended to deliver tACS phase-locked to the activity of the underlying brain region in real-time. This novel approach extracts phase and frequency from a segment of EEG, then forecasts the signal to control the stimulation. A careful tuning of the EEG segment length and prediction horizon is required and has been investigated here for different EEG frequency bands. The algorithm was tested on EEG data from 5 healthy volunteers. Algorithm performance was quantified in terms of phase-locking values across a variety of EEG frequency bands. Phase-locking performance was found to be consistent across individuals and recording locations. With current parameters, the algorithm performs best when tracking oscillations in the alpha band (8–13 Hz), with a phase-locking value of 0.77 ± 0.08. Performance was maximized when the frequency band of interest had a dominant frequency that was stable over time. The algorithm performs faster, and provides better phase-locked stimulation, compared to other recently published algorithms devised for this purpose. The algorithm is suitable for use in future studies of phase-locked tACS in preclinical and clinical applications.

## Introduction

Transcranial electrical stimulation (tES) has shown considerable promise for modulating brain activity in both preclinical and clinical applications (Nitsche and Paulus, 2000; Kuo and Nitsche, 2012; Dayan et al., 2013; Ruffini et al., 2013; Karabanov et al., 2016). Transcranial direct current stimulation (tDCS) is one tES modality with mounting evidence of efficacy as a treatment for an increasingly wide range of neurological or psychiatric disorders in recent studies and meta-analyses (Fregni et al., 2006, 2007; Nitsche et al., 2009; Brunoni et al., 2011; Brunelin et al., 2012; Kuo et al., 2014; Meron et al., 2015). Thanks to their safety, non-invasiveness, and low cost, tES techniques such as tDCS and transcranial alternating current stimulation (tACS) have the potential to become effective and inexpensive interventions for neurological and psychiatric illnesses characterized by abnormal brain activity. However, as these techniques are relatively new, the optimal parameters of stimulation are still poorly understood.

To date, the vast majority of tES studies have employed “open-loop” approaches, in which stimulation is applied without any input from the underlying target brain activity to be modulated. “Closed-loop” techniques, in which target brain activity via neurophysiological recording modalities like electroencephalography (EEG) is used as an input signal to continuously fine-tune the parameters of stimulation, are increasingly employed for invasive stimulatory therapies such as deep brain stimulation (DBS) (Rosin et al., 2011; Widge et al., 2017) or responsive neurostimulation (RNS) in the setting of epilepsy (Sun and Morrell, 2014). However, closed-loop techniques are far less commonly employed for non-invasive stimulation modalities such as tES, and indeed, few studies of such approaches exist in the current literature (for a recent review, see Karabanov et al., 2016).

Most tES research to date has focused on tDCS. However, the use of a constant current in tDCS stands in sharp contrast with the nature of physiological brain activity, which relies fundamentally on oscillatory behavior (Bishop, 1932; Buzsáki and Draguhn, 2004; Kaplan et al., 2005; Neuling et al., 2012; Womelsdorf et al., 2014). This mismatch between the waveforms of stimulation and target brain activity could potentially be preventing tDCS from exerting its desired effect. Recent tDCS studies have indicated that there is substantial variability in the response to constant-current stimulation both across individuals and even across sessions within a given individual (Wiethoff et al., 2014; Chew et al., 2015; López-Alonso et al., 2015). Although it is not clear why tDCS results are inconsistent, the oscillatory stimulation of tACS could potentially be more closely tuned to the neurophysiological processes of interest, and thus may be able to achieve more consistent or more potent effects in modulating brain activity for research or clinical purposes (Reato et al., 2010; Ali et al., 2013; Kutchko and Fröhlich, 2013).

Preclinical studies have shown tACS to be effective in modulating cortical excitability (Kanai et al., 2010; Zaghi et al., 2010; Wach et al., 2013) and in modulating frequency specific brain activities as recorded through EEG (Zaehle et al., 2010; Vossen et al., 2015). As with tDCS, the placement of the electrodes and the intensity of the stimulation are important parameters of stimulation for tACS (Kanai et al., 2008; Tecchio et al., 2013; Ruffini et al., 2014). However, tACS has additional parameters to be controlled: specifically, the frequency and phase of stimulation. These parameters likely require optimization in order for tACS to be used most effectively for the treatment of brain disorders (Karabanov et al., 2016). Such optimization will require both some knowledge about the frequency and phase of the target brain activity, and some method for using this information to regulate the frequency and phase of the stimulation itself.

The frequency of the tACS stimulation has been shown to be an essential parameter in targeting brain networks and functions, with several studies reporting that the effect of the stimulation is stronger when the frequency of the stimulation is matched to the underlying brain activity (Kanai et al., 2008; Feurra et al., 2011; Wach et al., 2013; Voss et al., 2014; Riecke et al., 2015; Cappon et al., 2016). In one well-cited example, tACS was demonstrated to be capable of inducing lucid dreaming during REM sleep—but only when applied at frequencies in a specific range from 25 to 40 Hz (Voss et al., 2014). Thus, the frequency-specific effects of tACS have the potential to provide stimulation that is tuned to underlying brain activity, and has potent effects in altering brain function.

Aside from frequency, the phase of stimulation is also proving to be an important parameter in determining the potency of tACS to modulate brain activity. The phase difference between the tACS stimulation and the target brain oscillations has been shown to be an important factor in determining how tACS modulates functions such as vision, hearing, motor activity, tremor, cognition, and working memory performance (Polanía et al., 2012; Brittain et al., 2013; Riecke et al., 2015; Chander et al., 2016; Guerra et al., 2016; Stonkus et al., 2016). In addition, it is not clear whether and to what extent the brain will naturally entrain to the external signal of tACS (Neuling et al., 2015; Witkowski et al., 2015; Chander et al., 2016). Previous studies show that there is either a very weak entrainment (Chander et al., 2016) or the entrainment is strong only at very close proximity to the tACS electrodes (Witkowski et al., 2015). In the absence of entrainment, successful phase-locking would require tACS to be continuously tuned to the underlying brain oscillations, in a “closed-loop” manner.

To study phase-associated effects of tACS, several different experimental designs have previously been used to avoid the challenges around providing phase-locked brain stimulation. External stimuli, such as auditory stimuli (Riecke et al., 2015) or visual stimuli (Polanía et al., 2012), can induce brain rhythms at known phase and frequencies, and can be used to provide an external benchmark for applying phase-locked brain stimulation. Alternatively, *post-hoc* analysis of brain recordings during the stimulation can determine the instantaneous phase of the stimulation compared to the brain activity (Neuling et al., 2015; Witkowski et al., 2015). However, these approaches are only applicable when the external sensory or motor oscillations may be used as a benchmark signal. For higher-order regions involved in cognition or emotion regulation, such external benchmarks may be unavailable. A more generally applicable approach would require (i) direct recording of oscillatory activity from the target brain region; (ii) a method for extracting the desired frequency and phase from the signal in real time, and (iii) a method for using this information in real time to control the tACS frequency and phase in an adaptive manner.

With these desiderata in mind, our objective is to develop a computationally efficient algorithm that can analyze brain oscillations obtained from EEG recordings, and use this information in real time to provide phase-locked tACS at a desired frequency band. To date, there has been relatively little published work on EEG-based phase-locked tES. One study sought to accomplish phase-locking through autoregressive (AR) modeling of the intracranial EEG signal (Chen et al., 2013). The inherent difficulty is that, in order to set the stimulation parameters, the future EEG signal must be predicted from a segment of past EEG. Precise forward modeling of all components of the EEG signal is challenging, and unlikely to be feasible with acceptable accuracy under real-time constraints. As an alternative approach, here we have investigated the performance that can be achieved by using a simple forward model of the EEG signal to select the tACS parameters, relying on frequent updates of this model to ensure close tracking of frequency and phase over time.

We sought to demonstrate the feasibility of this approach to achieve real-time, phase-locked tACS. Given that the amount of entrainment between the brain activity and tACS stimulation is not well understood, it is presently challenging to accurately test a phase-tracking algorithm on EEG data recorded while tACS is being applied. Therefore, we present here a proof-of-concept evaluation of the proposed approach on simulated and off-line EEG recordings. Demonstrating the feasibility and theoretical performance of this phase-locking approach is an essential first step in the development of a closed-loop phase-locked tACS system.

## Materials and methods

In overview, here we have developed and tested an algorithm that can forecast the dominant frequency and phase of an EEG recording within a band of interest. We test the performance of the algorithm on both synthetic signals and real EEG recordings obtained from 5 healthy participants. We sought to determine under what conditions and with what performance phase tracking could be achieved, focusing on the influence of frequency band, recording site, and algorithm parameters. Performance was compared to a previously proposed method based on AR modeling of the EEG (Chen et al., 2013).

### Phase-tracking approach

Our goal was to develop an algorithm that can deliver oscillatory brain stimulation output at a given frequency while maintaining a phase lock on the EEG activity at that frequency in real time. This is achieved by first analyzing the recorded EEG signal over a defined window of time, and then forecasting this signal into the future. The forecasting method relies on the assumption that in a small window of time, the phase and frequency of a dominant oscillation in the frequency band of interest of the recorded EEG signal will remain approximately stable. An EEG signal segment with duration D_past_ is used to forecast phase and frequency of the stimulation signal for a duration D_future_. This forecasting step is repeated continuously to achieve signal phase-locking over time. If the phase and frequency of the EEG signal are extracted from an adequately sized time window D_past_, an accurate forecasting of the EEG signal is hypothesized to be possible. Details of the algorithm are presented below.

#### Phase-tracking algorithm

**Figure 1:**
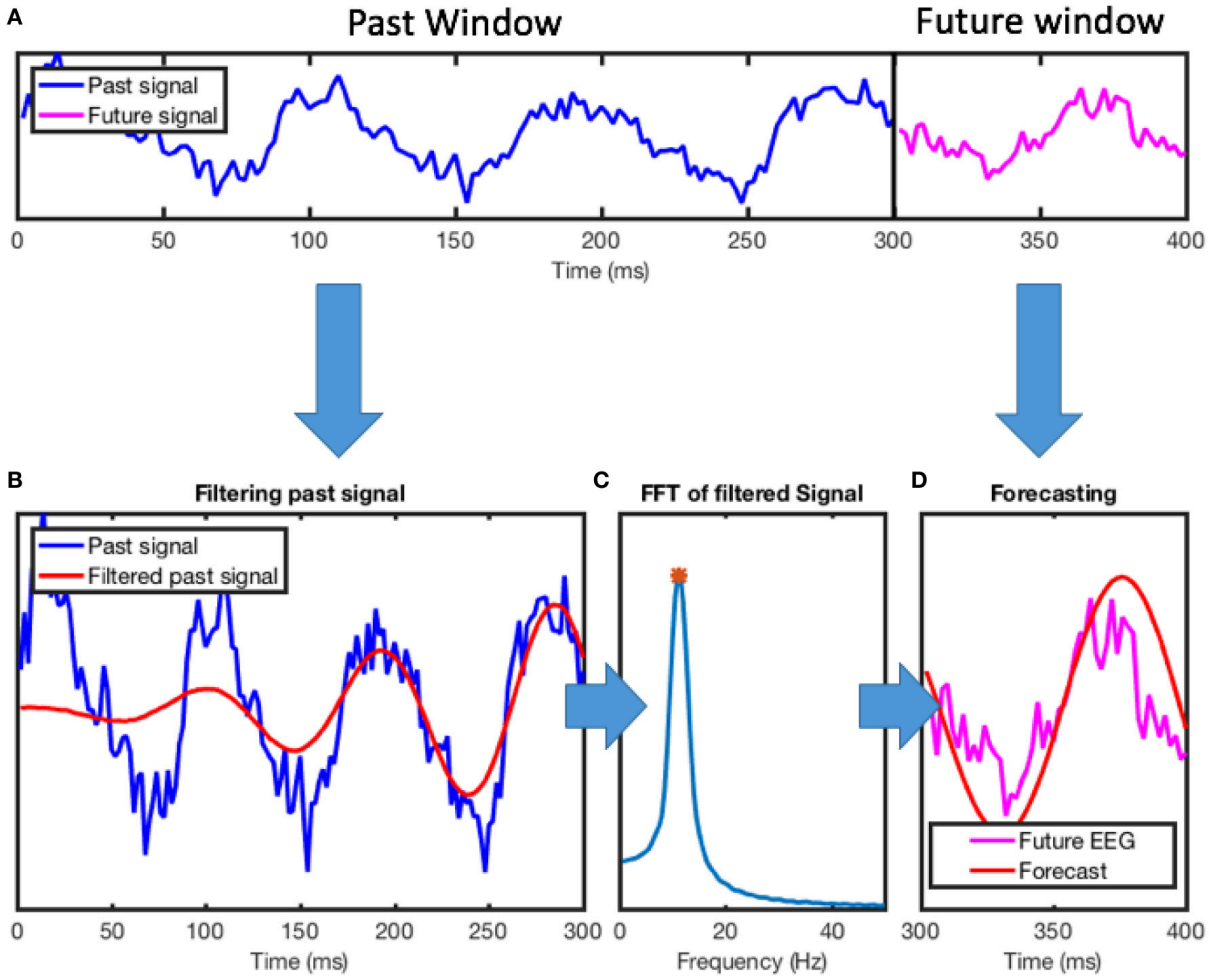
Overview of phase-tracking algorithm. **(A)** EEG recording from a healthy participant (electrode PO_Z_ in the standard 10–20 EEG montage). The algorithm uses a segment of the data (past window, D_*past*_) to forecast the signal into the future (future window, D_future_). Here, a 300 ms window of past EEG data was used to forecast 100 ms into the future. **(B)** The algorithm first uses a IIR band-pass filter to separate the signal that is within the frequency band of the interest. Here, the frequency band of interest is the alpha band (8–13 Hz). Note that due to the input of a small segment of the signal in this step, the filter introduces an artifact in the beginning of the segment. **(C)** The FFT of the filtered signal is then calculated. The FFT bin with maximum content is selected as the dominant frequency in the signal and its phase and frequency is extracted for forecasting. **(D)** Based on the estimated phase and frequency, a sine wave is used to forecast the signal. For performance evaluation, this forecasted signal may be compared to the EEG signal to determine the phase-locking value. IIR, infinite impulse response filter; FFT, fast Fourier transform.

The algorithm involves several steps that are performed sequentially as shown in Figure 1. First, the recorded EEG signal over a time window with duration D_past_ is bandpass filtered around the target frequency of interest; second, the Fast Fourier Transform (FFT) of this signal segment is calculated; third, the frequency and phase of the dominant component of the signal are calculated from the FFT; finally, using the calculated frequency and phase, the signal is forecasted for the duration D_future_.

The first step of the algorithm is to filter the data to extract the frequency band of interest. The algorithm uses a window of recorded EEG data with duration (D_past_) as the basis for forecasting a signal. The small sizes of the D_past_ would limit the order of the finite impulse response (FIR) filters, and therefore this approach cannot not be reliably employed. Instead, a 10th order elliptical infinite impulse response (IIR) filter is used to bandpass-filter the recorded data. The filter was designed using MATLAB “ellip” function to design a 10th order filter with 0.5 dB of passband ripple and 40 dB of stopband attenuation. To avoid floating point instability commonly encountered when the sampling frequency is much higher than the filtering frequencies, we convert the filter transfer function to second-order sections using MATLAB “ss2sos” function which are applied in a sequential manner using MATLAB “sosfilt” function (Figure 1B).

In the next step of the algorithm, to extract the phase and frequency components of the signal, its FFT is calculated based on the MATLAB “fft” function [based on FFTW (Frigo and Johnson, 2005)]. Zero-padding of the signal to ten thousand sample points is applied to decrease the bin sizes in the FFT, providing a higher frequency resolution through interpolation. The frequency of the signal is determined by the frequency of the FFT bin with the maximum magnitude; the phase of the signal is given by the angle component of its complex value. The algorithm uses a simple sine function to forecast the signal using the calculated phase and frequency parameters. To correct for the phase-shift due to filtering, a frequency-dependent correction factor is added to the phase calculated from the phase delay of the filter. Note that in an on-line implementation of this method, all instrumentation delays would need to be considered, but in the off-line analysis presented here these adjustments were not necessary.

#### Comparison algorithm

A benchmark algorithm is desirable to assess the performance of the present algorithm vs. previously published methods. Chen et al. (2013) used an AR method to forecast the EEG signal. As with our method, the EEG signal was first filtered using an IIR filter. However, in an approach diverging from our technique, the result was then used to train a 50th order AR model using the Yule-Walker algorithm. We used MATLAB “ar” function to train the AR model. The trained AR model was used to forecast the EEG signal into the future using MATLAB “forecast” function. As the method of Chen et al. was devised with similar objectives to those of our own work, this method serves as the comparator method for the purposes of evaluating the performance of our phase-tracking algorithm. For consistency and comparison purposes, we used the same D_past_ and D_future_ sizes when comparing the algorithm by Chen et al. to the algorithm proposed in this work. We also used the same filtering method for both algorithms. Chen et al., used a genetic optimization method to optimize the order and type of the filter. While their algorithm used only low order filters (order 1–2), here we have used stable 10th order IIR filters. This divergence in implementation from the previously reported method is considered to be acceptable, since it is expected to improve rather than hamper the performance of the comparison algorithm. This choice was made in order to reduce the number of factors that differ between the two methods.

### Evaluation data

The algorithms were tested on synthetic signals as well as previously recorded EEG recordings.

#### Synthetic signals

The use of a synthetic signal had two specific purposes, namely to investigate the effects of signal-to-noise ratio in a controllable manner, and to compare the FFT and AR approaches in a scenario where a ground truth was available. The synthetic signals were generated by adding Gaussian noise to a sine wave. The sampling frequency was selected to be at 500 Hz. We have selected a 500 Hz sampling frequency to ensure that aliasing is avoided; higher sampling rates are unlikely to contribute additional precision if the target EEG signals for phase-locking are in the much lower range from delta to gamma frequencies (i.e., <60–80 Hz), and might reduce the speed of the algorithm to the point of preventing real-time tuning. The signal-to-noise ratio of this synthetic signal was measured by taking the ratio of the power in the signal to the power in the noise.

#### EEG recordings

The algorithm was also tested on EEG recordings from 5 volunteers. Volunteers (5 female 42.2 ± 16.8 years, 4 right-handed) were recruited. EEG recordings were acquired for 5 min while the volunteers were sitting quietly with their eyes closed. The EEG data was recorded using ANT Neuro *eego Sport* device (ANT B.V., Enschede, The Netherlands), and the recordings were from 64 channels of EEG sensors positioned using the 10–20 EEG standard montage, via ANT Neuro Waveguard caps (ANT B.V., Enschede, The Netherlands). The sampling frequency was set to 2,000 Hz, and no in software filtering was applied during the recording. The data acquisition system included a hardware low pass filter with cut-off frequency of 524 Hz. After the recording, the data was down sampled to 500 Hz. Approval for the study, including the acquisition of EEG recordings from the volunteers, was given by the Research Ethics Board of the University Health Network.

### Performance evaluation

Both algorithms were implemented as functions in MATLAB 2015b. The functions took in an EEG signal vector with length D_past_, and returned the forecasted signal vector with length D_future_. Note that the forecasted signal is simply a sinusoid, which corresponds both to the estimated future EEG and to the desired tACS waveform. This function was suitable to be used for real-time application; however, for testing purposes we developed an offline routine to simulate the performance of the algorithm using pre-recorded data. For computation time measurements, a MacBook Air 1.8 GHz Inter Core i5 2012 and an AsusTek AMD A10-6700 3.7 GHz were used.

A moving window approach was used to test the algorithms offline. The pre-recorded data was sampled using a window with size D_past_ and the window was moved with the step size D_future_. At every step the algorithms were applied to forecast the signal. The forecasted signal was saved for further comparison with the original signal.

Different frequency bands were used to test the performance of the algorithms. Established EEG frequency bands were used for frequencies below 20 Hz [delta (2–4 Hz), theta (4–8 Hz), alpha (8–13), and low beta (13–20 Hz)]. For frequencies above 20 Hz, the beta and gamma bands were split into smaller bands of 10 Hz (20–30, 30–40, and 40–50 Hz). Phase tuning to a dominant frequency becomes less meaningful when the bands are wide, due to the potential for multiple dominant peak frequencies within each band. Frequencies above 50 Hz were left outside the scope of this study because of the decreasing power in the EEG signal at those frequencies.

To compare execution time between the two algorithms, each iteration was timed through a MATLAB script. The run time for each iteration was measured 100 times for a range of input window sizes, D_past_, from 250 to 2,000 ms, while the D_future_ window size was set at 20 ms.

#### Performance metrics

For comparison purposes, the EEG signal was filtered using the bandpass filter to extract a dominant frequency, which should ideally be matched exactly by the forecasted signal or stimulation. The phases of the forecasted signal (φ_forecast_) and the EEG signal (φ_EEG_) were calculated using Hilbert transformation and compared using the Phase Locking Value (PLV). The PLV was used to evaluate the performance of the proposed algorithm. PLV is a value between 0 and 1, calculated using Equation (1).

(1) $$\begin{array}{l} PLV=\left\|\frac{1}{N}\sum_{i=1}^{N}e^{i\left(\varphi_{EEG}-\varphi_{forecast}\right)}\right\| \end{array}$$

In addition to this assessment of phase-locking performance, the algorithms were also timed to measure their associated computational costs, in order to assess their suitability for real-time use. The computation-time measurements were repeated 100 times to provide a more accurate indication of the delay of each algorithm in processing the input data and generating an output signal. In addition, we also tested the algorithms' speeds at different past window sizes to evaluate the relationship between window size, phase-locking performance, and computation time.

## Results

### Synthetic signal

**Figure 2:**
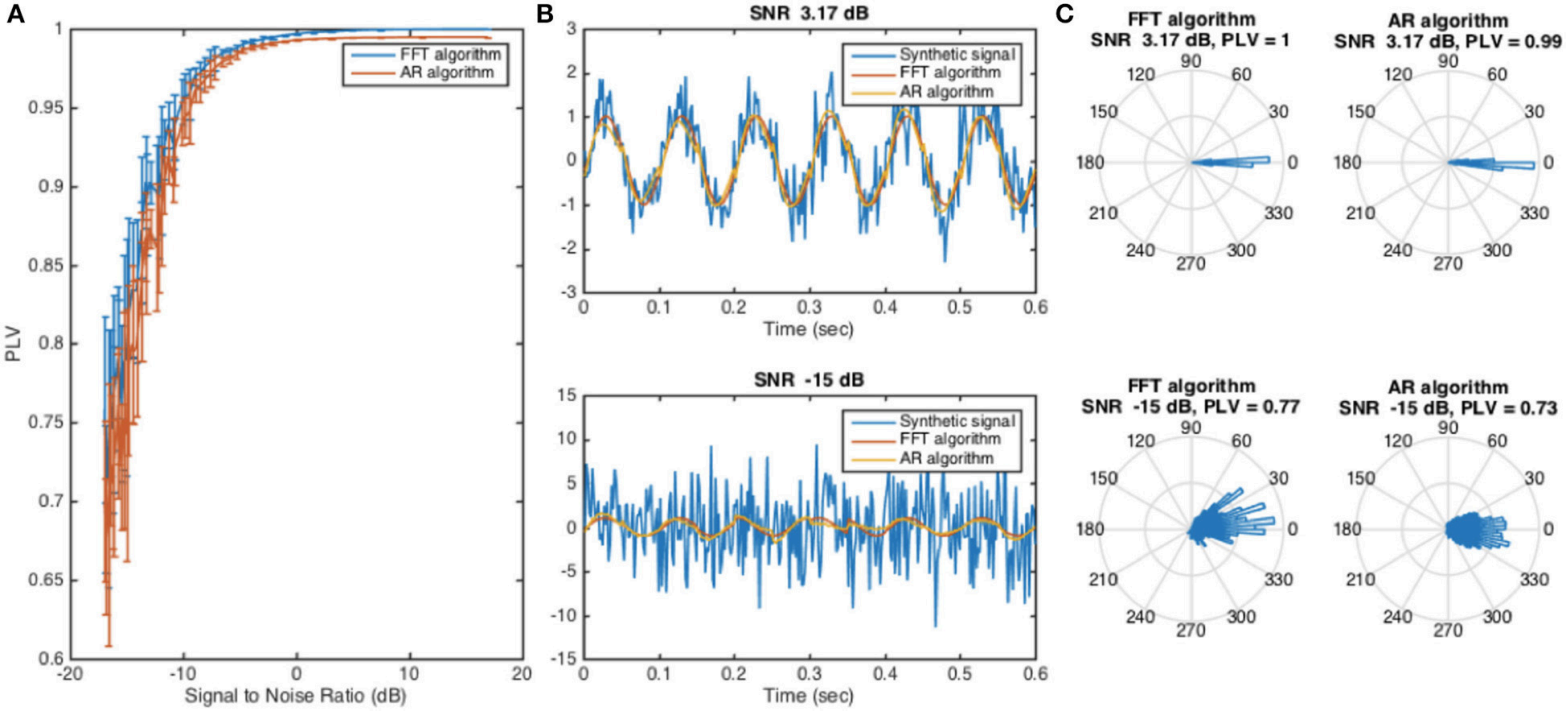
Evaluating the FFT algorithm and AR algorithm when applied to synthetic signal at 10 Hz. **(A)** The phase locking value was used as a metric of algorithm performance. As the signal-to-noise ratio decreased, the performance of the algorithms also decreased. The simulation was repeated 10 times; the value and the error bars at each point were calculated by taking the mean and the standard deviation of the 10 measurements. **(B)** Two synthetic signals, one with high signal-to-noise ratio and one with low signal-to-noise ratio, were selected and are presented along with the forecasted signal. **(C)** Rose plots of phase difference between the ideal forecasted signal and the forecasted signal from the algorithms illustrate phase-locking performance of the FFT and AR algorithms under high and low SNR conditions on the 10 Hz synthetic signal.

The algorithms were first tested on synthetic signals that were generated using a 10 Hz sine wave with added Gaussian noise. The performance of the two algorithms was comparable (Figure 2), and ranged from PLVs of 0.6 to 1. The performance dropped as the amount of noise added to the signal increased. In these tests, D_past_ was set to be 300 ms and D_future_ was set at 50 ms. Changes in these parameters can change the performance of the algorithms (see Section Optimizing Window Sizes for Stimulation Using Actual EEG Data); however, the trend of change with SNR remains the same.

### Computation time

**Figure 3:**
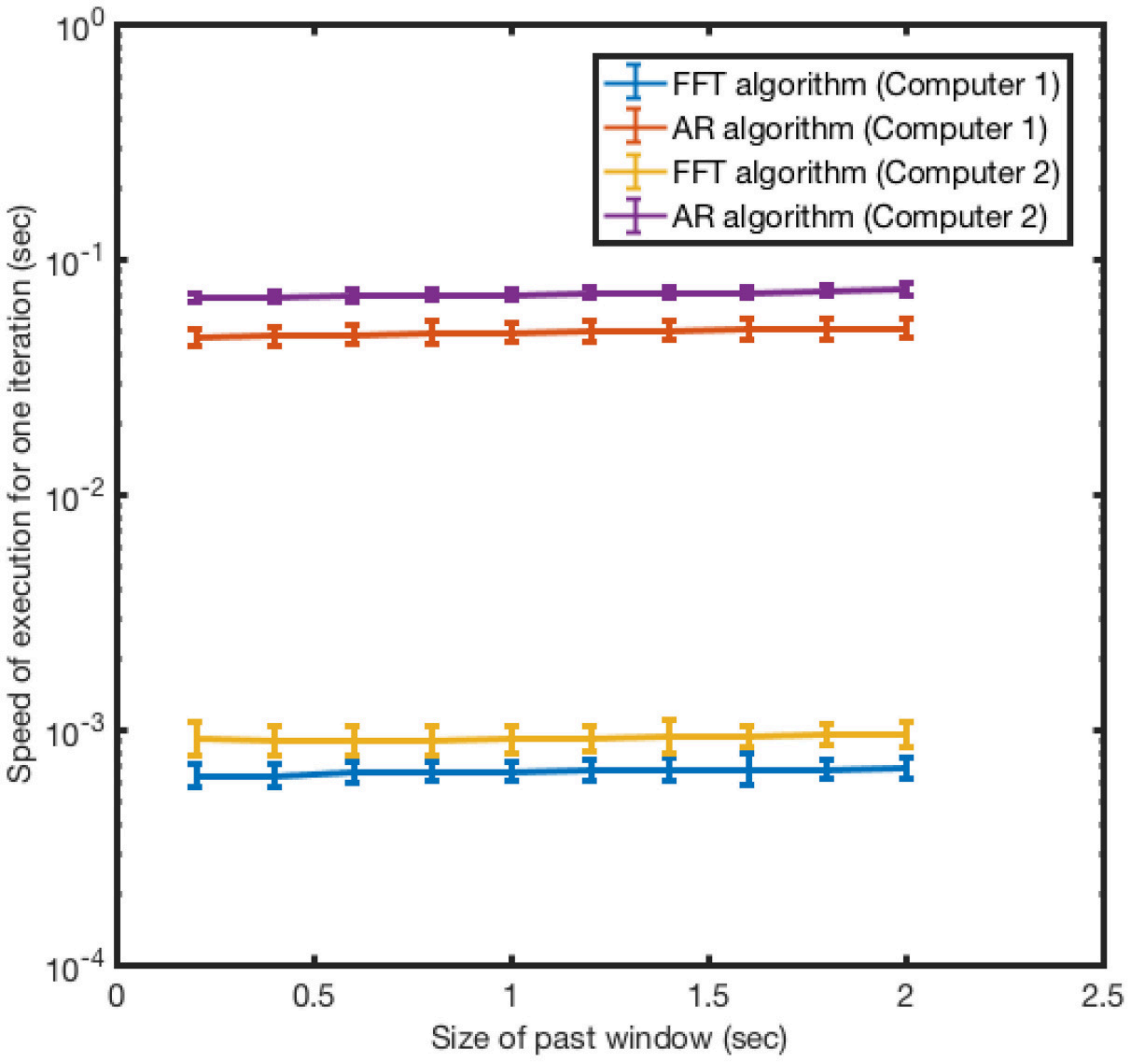
Speed of execution of iteration for FFT algorithm and AR algorithm. The time for each iteration was measured 100 times for a range of input window sizes D_past_ from 250 to 2,000 ms. The D_future_ window size was set at 20 ms. The speed and the error bars for the speed are calculated by taking the mean and standard deviation of the 100 measurements. Computer 1 is a MacBook Air 1.8 GHz Inter Core i5 2012 and computer 2 is an AsusTek AMD A10-6700 3.7 GHz.

As only a sufficiently fast algorithm is suitable for real-time applications, we next assessed the computation time for the FFT and the AR algorithms. The present algorithm based on FFT proved to be approximately two orders of magnitude faster than the AR algorithm when run on the same hardware (Figure 3). For example, for a D_past_ of 400 ms, the FFT algorithm required 0.68 ms of computation time to forecast the signal, compared to 72 ms for the AR algorithm. Increasing size of the input window D_*past*_ over the range 250–2,000 ms only slightly increased the computation time for either algorithm. In all cases, the run time for the FFT-based algorithm was less than 1 ms. It is important to note that the size of the forecast window D_future_ does not affect the run time of the algorithm for one step; however, it controls the number of steps the algorithm needs to run.

### Optimizing window sizes for stimulation using actual EEG data

**Figure 4:**
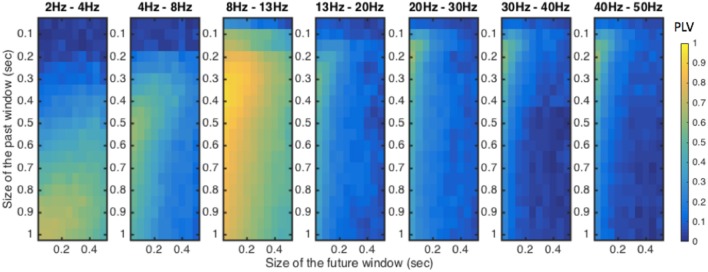
Performance of the algorithm across different future and past window sizes in different frequency bands. The algorithm was evaluated at window sizes ranging 50–500 ms for future window and 50–1,000 ms for past window from recording site PO_Z_ (10–20 EEG system). The PLV value was used to evaluate the performance of the algorithm across these ranges in each EEG frequency band.

We next assessed how the sizes of the past and future windows affected the performance of the algorithm. We explored the relationship between window size and phase-locking performance across a range of frequency bands commonly studied in EEG recordings. For each band, the performance of the algorithm was calculated at a range of input window sizes from 50 to 1,000 ms, and across a range of output window sizes from 50 to 500 ms. The results of this performance evaluation are illustrated in Figure 4, using recorded EEG data for one recording site (PO_*Z*_ in the standard 10–20 EEG montage) in one representative participant. For all frequency bands, performance universally improved as the size of future window decreased. However, the optimal past window size proved to be dependent on the frequency band of interest, ranging from ~200–300 ms at higher frequency bands to 900–1,000 ms at lower frequency bands. Thus, determination of the optimal window size is dependent on which EEG band contains the physiological signal of interest for modulation.

### Phase-locking performance on actual EEG signal across individual participants

**Figure 5:**
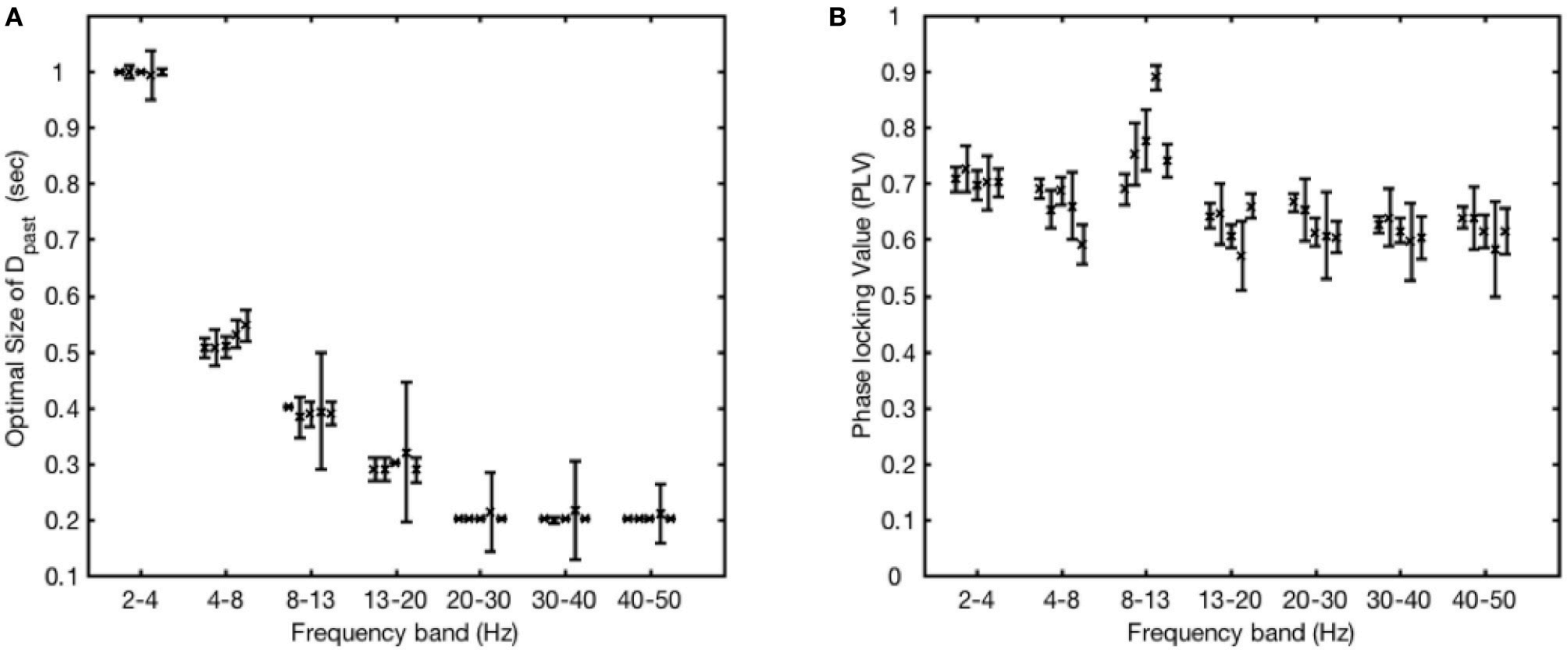
Performance of the algorithm at different frequency bands applied to EEG data recorded from 5 different participants. **(A)** Data obtained across all 64 channels of EEG recordings were used to calculate the optimal past window size D_past_ for each participant (participant plotted as adjacent points in each band). The points and the error bars for each participant are calculated by taking the mean and standard deviation of the optimal window size as determined over the 64 channels. **(B)** Using the optimal past window size for each channel, participant and frequency band, the performance of the algorithm was calculated in terms of phase-locking value (participants plotted as adjacent points in each band). The data points and their error bars are calculated by taking the mean and standard deviation over all the channels for each participant and frequency band.

Using the same window optimization strategy, we next determined the optimal past window across all 64 channels of EEG recordings obtained from the 5 volunteer participants (Figure 5). The optimal window size remained consistent across all participants when phase locking to any given frequency band (Figure 5A). In addition, the performance of the algorithm proved to be similar across participants, while the performance was best when phase locking to the 8–13 Hz frequency band (Figure 5B). An ANOVA showed a significant difference between frequency bands [*F*_(6, 28)_ = 11.59, *p* = 1.666E-6], and *post-hoc* pairwise *t*-tests showed a significant difference between 8 and 13 Hz and each of the other groups (*p* < 0.01 for each group, Bonferroni-corrected for multiple comparisons) except 2–4 Hz (n.s.).

**Figure 6:**
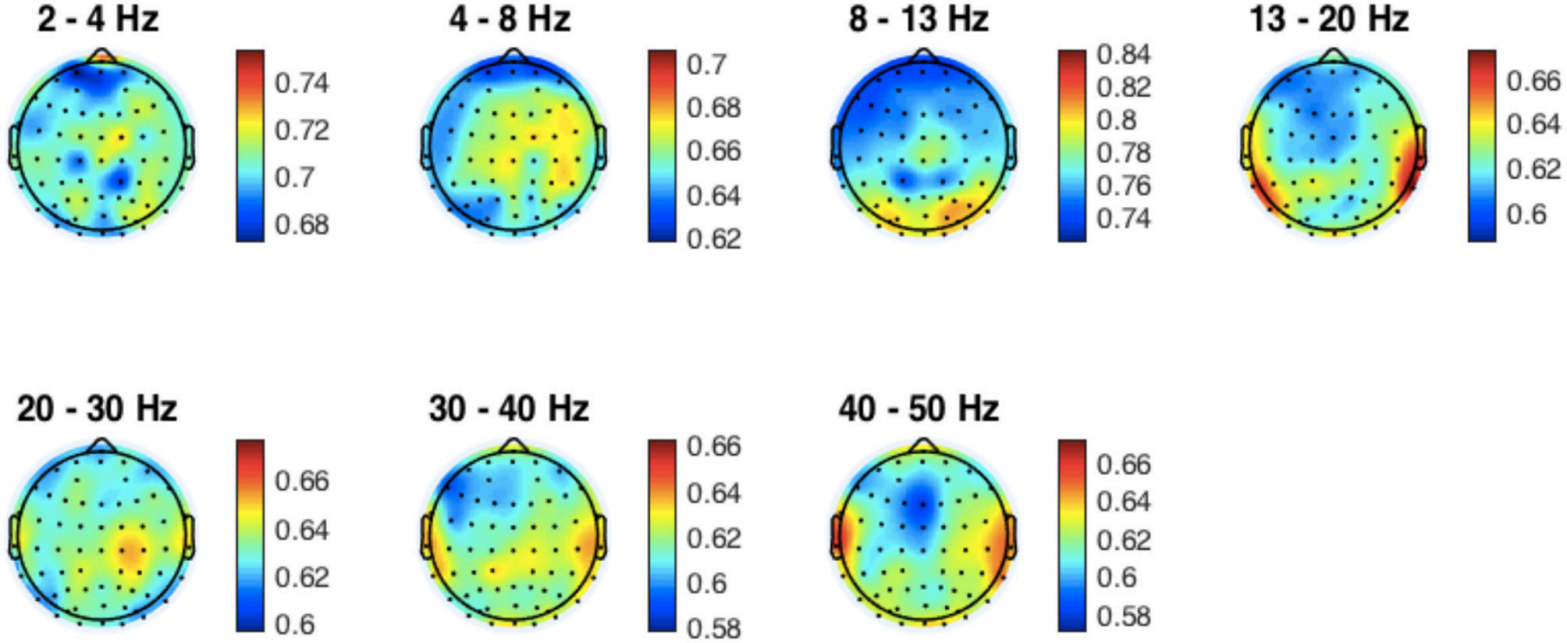
Phase-locking performance of each channel across individuals. Mean PLV values across individuals are mapped on to the standard head model at each EEG frequency band. Performance was consistent across sites and bands, with slightly better performance in the alpha band for occipital electrodes and slightly better performance in beta and gamma bands for temporal and lateral parietal sites.

We next assessed the performance of the FFT-based algorithm across the array of sensors in the 64-channel EEG montage. For this analysis, the phase locking performance was tested for EEG recordings acquired at each of the 64 different EEG sites, using the optimal values of D_past_ obtained from Figure 5A. For each EEG sensor, the PLV values calculated in each participant were averaged, and a map of these average PLV values was created (Figure 6). This map provides an indication of which locations provided recordings most suitable for phase locking to each frequency band. In general, the PLV variations between sites were comparatively minor, with performance overall in the 0.6–0.7 range across all sites and bands, varying by no more than 0.06 to 0.1 between the best and worst site at any given frequency band. Considered topographically, for occipital electrodes, phase locking performance was best in the alpha band (8–13 Hz), while for parietal electrodes, phase locking performance was best in the beta (13–20 Hz) and gamma (30–40 Hz, 40–50 Hz) frequency bands.

### Evaluating AR and FFT algorithms applied to the recorded EEG

**Figure 7:**
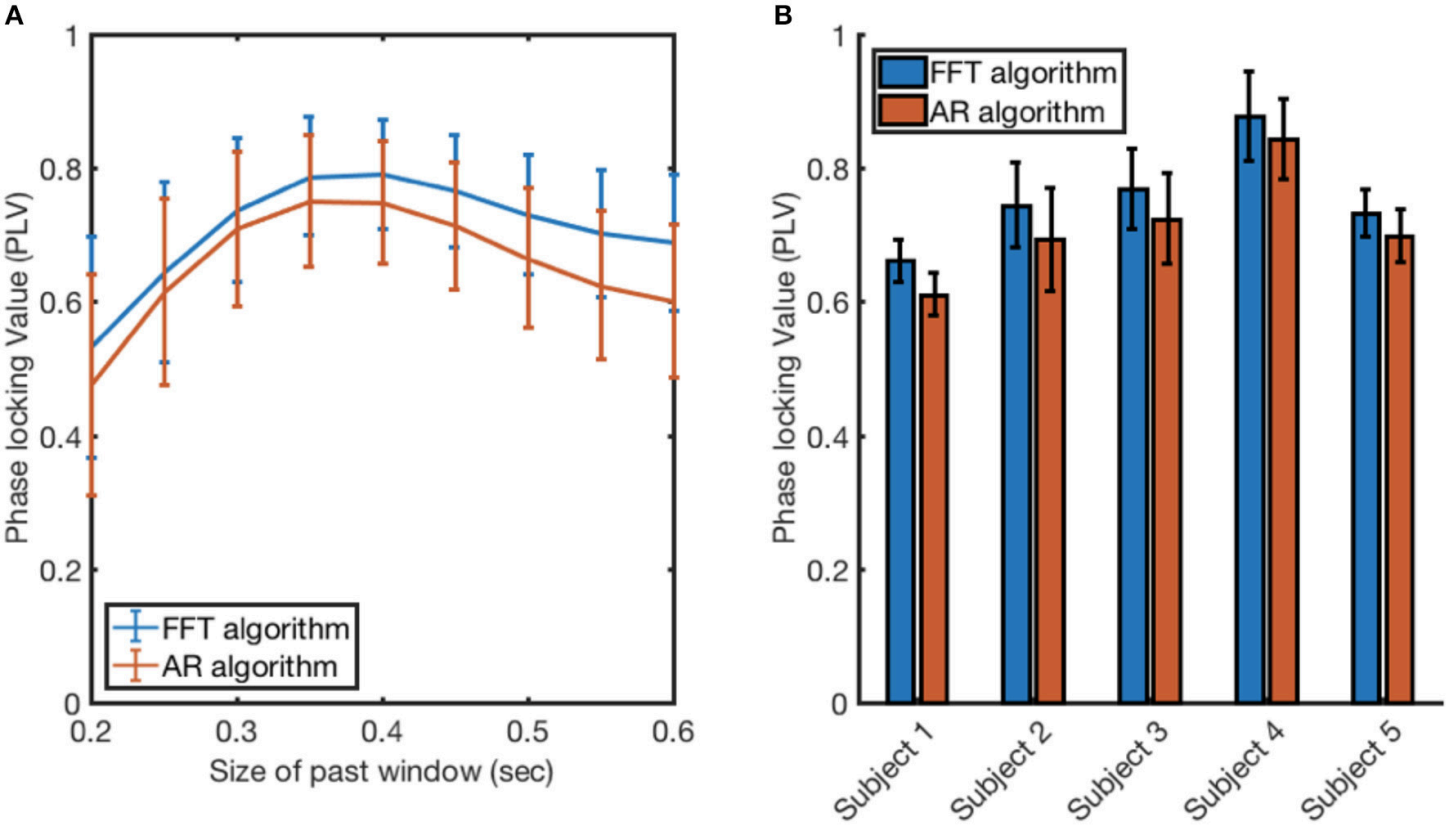
Evaluating the FFT algorithm and AR algorithm when applied to EEG recording at 10 Hz. **(A)** The size of the past window was varied between 0.2 and 0.6 s to find the optimal past window size for both algorithms. The value and the error bars at each point were calculated by taking the mean and the standard deviation of the PLV measurements across the 5 participants. **(B)** The performance of each algorithm measured through PLV when applied to EEG recorded from 5 participants. The value and the error bars at each point were calculated by taking the mean and the standard deviation of the PLV measurements across 64 electrodes.

AR and FFT algorithms were tested on EEG recordings to further compare their performances when phase tracking to alpha oscillations (8–13 Hz). First, the optimal size of past window for both algorithms was shown to be at 0.35 s (Figure 7A). Thus, the size of the past window was set to 0.35 s, and the algorithms' performance was compared across the participants (Figure 7B). The two algorithms' performances achieve comparable results, with the FFT algorithm performing slightly better in more than 99% of the electrodes. A three-way ANOVA analysis was conducted to examine the difference in the performance of the algorithms while controlling for the effects of electrodes and participants. There was a significant difference in the performance of the algorithms [*F*_(1, 571)_ = 133, *p* < 0.001].

### Effects of variability of the peak frequency and power of the frequency band on phase-locking performance

We investigated how the presence or absence of a dominant frequency that is stable over time can affect the performance of the FFT-based algorithm. First, using the optimal past window size for each channel, participant and frequency band (Figure 5A), the performance of the algorithm was calculated in terms of phase-locking value. Then, the variability of the peak frequency was calculated by taking its standard deviation; peak frequency values were taken from each window over the signal duration.

**Figure 8:**
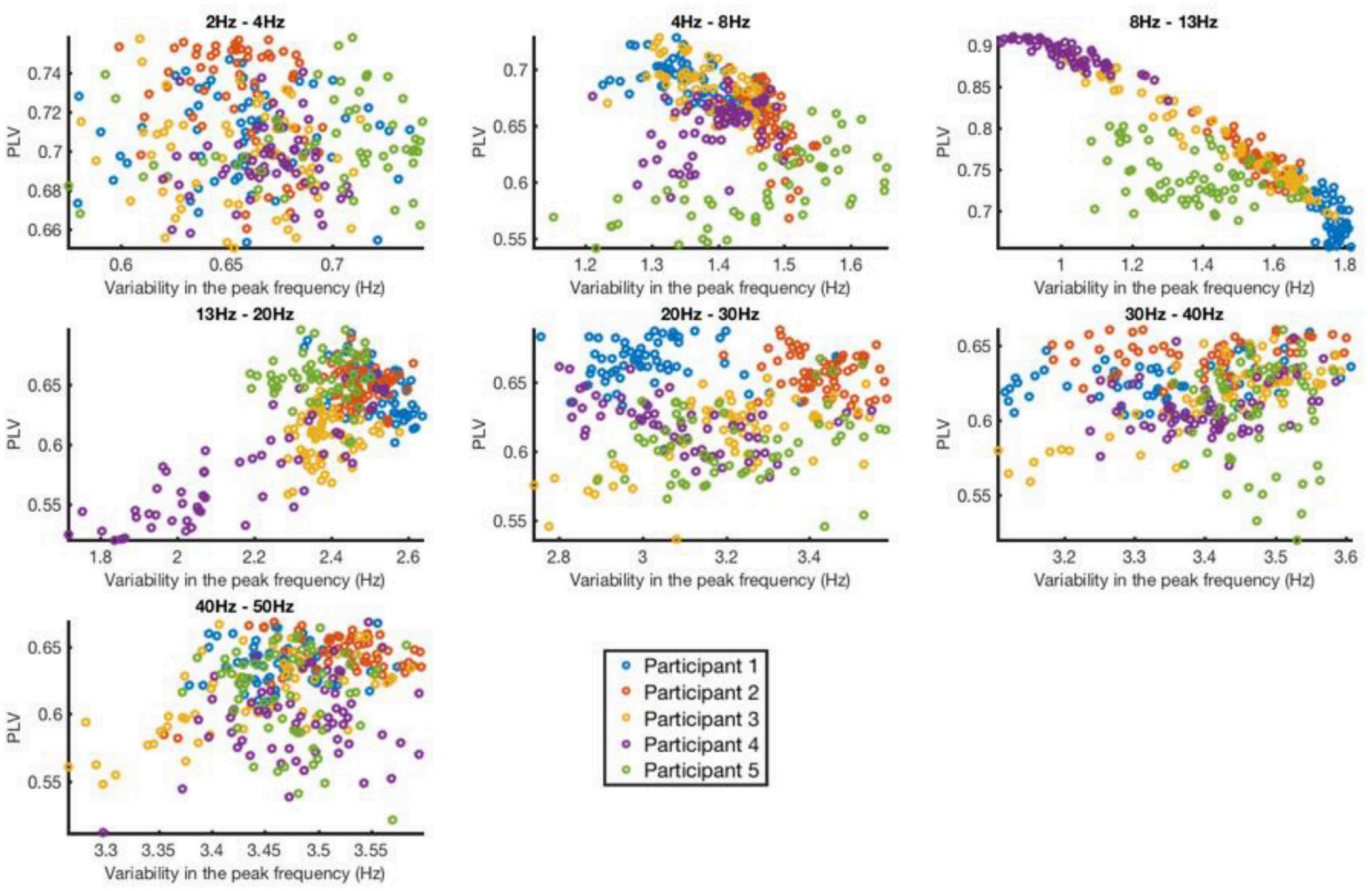
Phase locking value vs. variability in the peak frequency for each channel and participant in 7 different frequency bands. Each dot represents the data from a given channel and participant.

When phase locking to the alpha frequencies (8–13 Hz), it was found that the FFT-based algorithm performed better when the signal had smaller variability in the peak frequency, as might be expected (Figure 8). Notably, different individuals had rather different degrees of variability in peak frequency, resulting in some variability in the final PLV performance across individuals. However, in all participants, the algorithm achieved PLV values above 0.5, and in some participants achieved PLV values in the 0.8–0.9 range. While a strong negative relationship was observed between the average peak frequency variability and the average PLV in the Alpha band (8–13 Hz), no other frequency band exhibited such strong results. Overall, there was no consistent significant correlation between the average peak frequency variability and the average PLV (Table 1).

**Table 1:** Correlation between phase locking value and variability in the peak frequency.

	**Participant 1**		**Participant 2**		**Participant 3**		**Participant 4**		**Participant 5**	
**Bands**	***R*^2^**	***p***	***R*^2^**	***p***	***R*^2^**	***p***	***R*^2^**	***p***	***R*^2^**	***p***
2–4 Hz	<0.01	0.74	0.19	<0.01	0.03	0.17	0.80	<0.01	0.01	0.44
4–8 Hz	0.28	<0.01	0.07	0.04	0.35	<0.01	0.74	<0.01	0.40	<0.01
8–13 Hz	0.46	<0.01	0.08	0.02	0.96	<0.01	0.01	0.53	0.14	<0.01
13–20 Hz	0.49	<0.01	0.33	<0.01	0.03	0.20	0.84	<0.01	0.01	0.55
20–30 Hz	0.02	0.27	0.50	<0.01	0.36	<0.01	0.74	<0.01	0.01	0.53
30–40 Hz	0.14	<0.01	0.59	<0.01	0.61	<0.01	0.94	<0.01	0.01	0.36
40–50 Hz	0.05	0.09	0.75	<0.01	0.46	<0.01	0.89	<0.01	0.05	0.07

**Figure 9:**
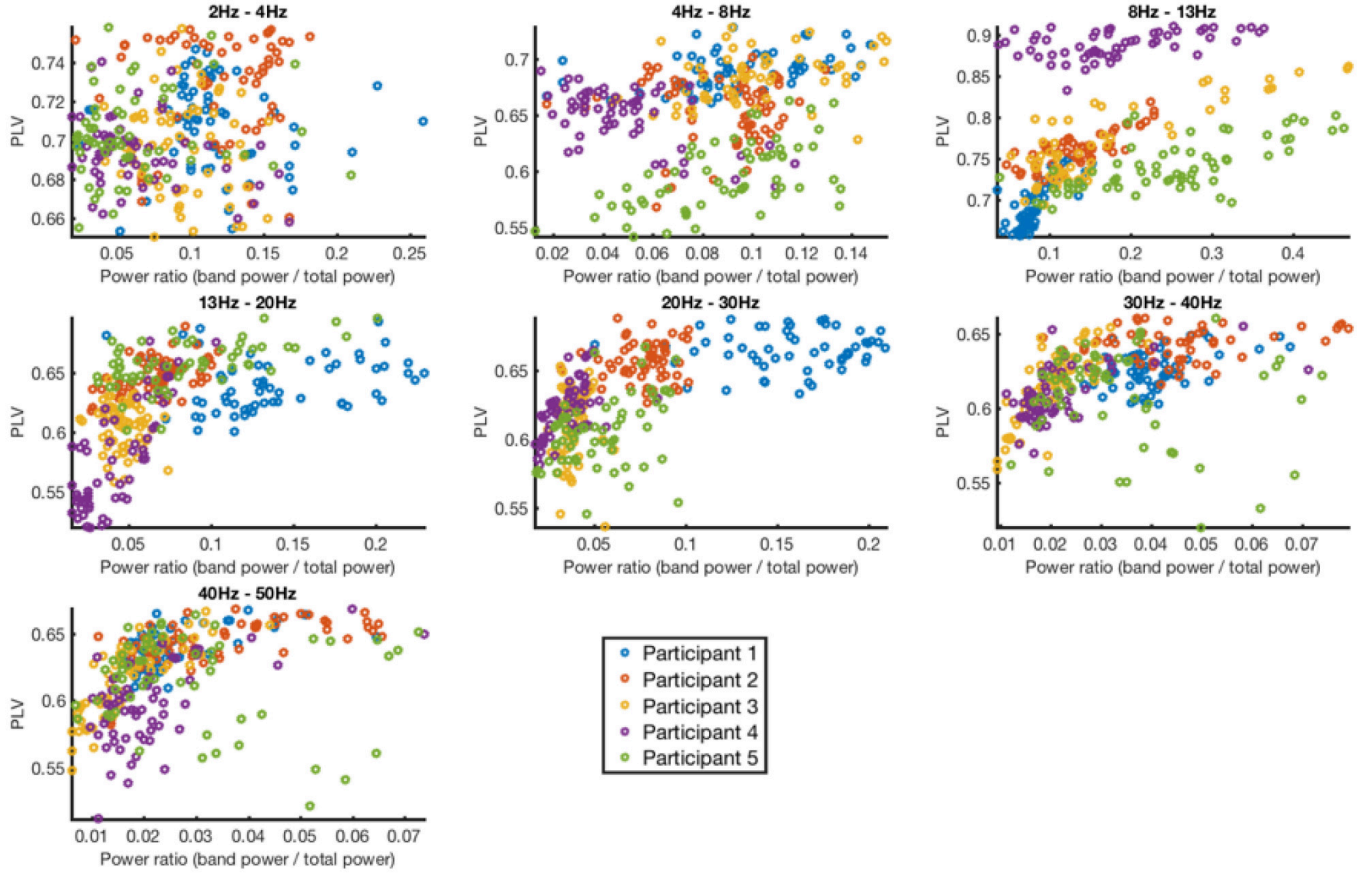
Phase locking value vs. band power for each channel and participant in 7 different frequency bands. Each dot represents the data from a given channel and participant.

Lastly, we investigated the relationship between the power of the frequency band of interest and the PLV (Figure 9). The power was quantified by measuring spectral power of the target frequency band divided by the power of the overall signal. The band powers did not show strong correlation with the PLV (Table 2).

**Table 2:** Correlation between phase locking value and power ratio.

	**Participant 1**		**Participant 2**		**Participant 3**		**Participant 4**		**Participant 5**	
**Bands**	***R*^2^**	***p***	***R*^2^**	***p***	***R*^2^**	***p***	***R*^2^**	***p***	***R*^2^**	***p***
2–4 Hz	0.03	0.14	0.34	<0.01	0.01	0.52	0.19	<0.01	0.01	0.54
4–8 Hz	0.17	<0.01	0.18	<0.01	0.06	0.04	0.26	<0.01	0.27	<0.01
8–13 Hz	0.52	<0.01	0.25	<0.01	0.80	<0.01	0.39	<0.01	0.45	<0.01
13–20 Hz	0.09	0.01	0.17	<0.01	0.02	0.26	0.59	<0.01	0.40	<0.01
20–30 Hz	<0.01	0.84	0.28	<0.01	<0.01	0.63	0.31	<0.01	0.08	0.02
30–40 Hz	0.30	<0.01	0.20	<0.01	0.62	<0.01	0.23	<0.01	0.16	<0.01
40–50 Hz	0.46	<0.01	0.19	<0.01	0.67	<0.01	0.23	<0.01	0.13	<0.01

## Discussion

Here we have presented a new approach for phase-locking tACS brain stimulation to an EEG signal in a manner that is fast enough to enable real-time, “closed-loop” phase-locked tES. Algorithms suitable for this purpose should be able to achieve good phase locking performance, while still being sufficiently fast for real-time implementation. To this end, a simple algorithm is desirable because it has fewer parameters to be optimized and thus can have a faster speed of execution. On the other hand, a simple algorithm is unlikely to be able to model all components of the complex EEG sufficiently well to forecast it more than a few milliseconds in the future, thus creating a need for frequent updating of the stimulation parameters.

Offline methods such as the complex wavelet transform (CWT) (Adeli et al., 2003; Valde et al., 2004) and the Hilbert-Huang transform (HHT) (Bajaj and Pachori, 2012; Lin and Zhu, 2012) have previously been used to extract phase and frequency information from EEG data. These methods, although very powerful for describing frequency and phase information in non-stationary signals like EEG, are not suitable for the delivery of phase-locked tACS in real-time, closed-loop applications. Their limitation arises because the analysis of non-stationary data is difficult to reconcile with the task of forecasting a future signal. Non-stationary analysis methods assume that the signal's properties will change over time, whereas the act of forecasting assumes that the system's dynamics will remain stable for a certain length of time. Although the EEG is highly non-stationary, the nature of the forecasting task with closed-loop tACS nonetheless requires that over at least a short time-period, an assumption of stationarity be made, thus rendering methods such as the HHT less well suited to real-time, adaptive closed-loop stimulation. The method proposed here assumes stationarity over short periods of time only and relies on frequent updates (made possible by a computationally efficient model) to adapt to a non-stationary signal; in this manner, forecasting is possible while still adjusting to changes in the signal dynamics over time.

In this work, we used an FFT-based algorithm to explore the tradeoffs between algorithm simplicity and speed on one hand, and phase-tracking performance on the other hand. The FFT-based algorithm was tested using both synthetic signals and recorded EEG. The algorithm was compared to the previously published algorithm by Chen et al. (2013) and was determined both to be faster (by up to 2 orders of magnitude) and to achieve a slightly tighter phase-locking as evident in higher PLVs. For synthetic signals, both algorithms performed well when the noise level was low, while performance dropped as noise level increased. The FFT-based algorithm works based on the assumption that the frequency of the signal of interest is stationary for short periods of time; thus, it can be estimated through FFT analysis. While the EEG signal is highly non-stationary, we found that this simplifying assumption can nonetheless lead to high PLV results if the algorithm parameters are tuned to the frequency band of interest.

The run times for the AR model appeared to be longer than the forecasting window, making the method inapplicable to real-time phase-locked tACS; although a faster processor could remedy this issue, such a processor may not be suitable for use in some clinical settings (e.g., at-home or portable tACS devices). As such, the FFT-based algorithm may be better suited for eventual implementation in small, inexpensive, home-based stimulators, without any sacrifice of performance.

Based on the FFT algorithm performance, it takes about 1 ms of computation time to predict 25 ms into the future. Considering the use of this method in a closed-loop phase-locked tACS device, the delays in the closed loop system should not be larger than 24 ms. Given steady recent progress in the development of new tACS devices, this constraint is not expected to be difficult to overcome. On the other hand, the past window needs to be considerably longer than the forecasting window (see Figure 4). This result implies that, in order to achieve reasonable stimulation duty cycles, it will be necessary to record and stimulate at the same time. The applicability of the method proposed here will therefore be contingent on being able to effectively remove the sinusoidal stimulation artifact from the recordings. Methods such as template subtraction (Voss et al., 2014), synthetic aperture magnetometry (Soekadar et al., 2013), saw tooth tACS (Dowsett and Herrmann, 2016), and amplitude modulated tACS (Witkowski et al., 2015) have been used to remove artifacts during tACS stimulation. If our analysis had revealed that a longer forecasting with shorter past windows was possible, we could have concluded that alternating stimulation and recording was possible, thus avoiding the issue of artifact removal. However, our results show that the artifact removal step will indeed be essential.

Our evaluation of the FFT-based algorithm included analyses performed to determine the optimal parameters for the past and future windows D_past_ and D_future_, when phase-locking to different frequency bands (Figures 4, 5). Importantly, in these analyses, the optimal parameters were found to be consistent between individuals, a finding which supports the generalizability and clinical relevance of the proposed method. The algorithm performed maximally when phase-locking to the alpha band. A slightly lower performance in the gamma band compared to the alpha band was to be expected, because gamma is a broader band and less likely to have a well-defined peak frequency that the phase-tracking algorithm can lock onto. Nonetheless, performance met or exceeded a PLV of 0.6 across all frequency bands from delta to high gamma (Figure 5), such that the FFT-based algorithm still appears suitable to be used across a variety of different frequency bands of interest.

Testing on the EEG data recorded from 5 healthy participants revealed only minor variability in performance of the algorithm both among locations of EEG recording and individuals. The observed variability can be due to multiple reasons, including noise level during the recording and frequency differences among the regions of the brain and individuals. Our analysis shows that there is a relationship between the variability of the dominant frequency component in the alpha band (8–13 Hz) and the performance of our algorithm. However, this relationship was not visible in other frequency bands. Further testing may need to be done to discover how this variability will impact performance during more complex cognitive tasks.

The feasibility of real-time phase-locking of tACS stimulation to the underlying EEG signal opens up a new domain of potential studies in the field of non-invasive brain stimulation. At present, variability of response to tES is considerable both across individuals and across sessions within a given individual (Wiethoff et al., 2014; Chew et al., 2015; López-Alonso et al., 2015). Moreover, the potency of tACS in modulating any given brain function increasingly appears to be dependent on whether the frequency of stimulation matches the frequency of the target brain process of interest (e.g., Voss et al., 2014). However, at present, it remains an open question as to how phase-locking might affect the potency of tACS, or indeed whether “closed-loop” phase-locking might help to reduce the variability of tES itself.

The present study was intended to demonstrate proof-of-concept and feasibility for an FFT-based, real-time algorithm for phase-locked tACS. Considering the prospects of closed-loop tACS, a demonstration of phase-tracking on off-line recordings is a necessary first step. The brain's response to tACS, in terms of phase alignment, is currently poorly understood (Neuling et al., 2015; Witkowski et al., 2015; Chander et al., 2016). Characterizing the performance of a phase-tracking algorithm in the absence of an adaptive brain response will provide a frame of reference for future closed-loop experiments. The implementation of the method in a closed-loop experiment is needed next to demonstrate its performance in the presence of stimulation artifacts and possible entrainment of brain activity to the stimulation.

Limitations of the present study include a relatively small sample size and the need for further study of the method during the performance of behavioral tasks, rather than in the simple case of the eyes-closed resting state. Such future work will help to clarify the effect of in-phase vs. out-of-phase stimulation on motor, sensory, and cognitive functions of interest. Further, the effect of tACS on the EEG has not been considered in this study. It is anticipated that the EEG signal may change in response to the effect of tACS (Karabanov et al., 2016), and the effect of this EEG response will be observed once this algorithm is implemented in a closed-loop EEG-tACS system.

Future systematic studies of the role of phase-locking in tES will require an apparatus capable of maintaining stimulation that is phase-locked (or anti-locked) to the underlying brain activity with reasonable accuracy, for prolonged periods of time, adaptively and in real time. The FFT-based algorithm of the present study offers a viable approach for doing so, maintaining PLV levels that match or exceed previous approaches while incurring markedly lower computational burdens, and thus allowing faster adaptation to follow the underlying EEG signal. The FFT-based algorithm may thus enable more detailed study of a new and potentially important parameter of tES: namely, the role of phase-matching between the neuromodulatory tES input and the underlying EEG signal of interest.

## Conclusion

As the field of tES continues to improve its understanding of how stimulation parameters (frequency, intensity, waveform, montage) interact with underlying brain physiology, the role of phase-locking between the stimulator and the target brain activity will no doubt become clearer. A necessary precondition for progress on this issue is the availability of a technique for maintaining phase synchrony between the stimulator and the brain in real time. It is hoped that the algorithm presented here, along with other similar methods, will facilitate work on this issue and ultimately yield more effective tES methods, for both preclinical and the clinical applications.

## Author contributions

All authors listed, have made substantial, direct and intellectual contribution to the work, and approved it for publication.

### Conflict of interest statement

The authors declare that the research was conducted in the absence of any commercial or financial relationships that could be construed as a potential conflict of interest.
